# Supplementary material for: RXR Agonist V-125 Induces Distinct Transcriptional and Immunomodulatory Programs in Mammary Tumors of MMTV-Neu Mice Compared to Bexarotene
Source: Biomedicines. 2025 Dec 30;14(1):80. doi: 10.3390/biomedicines14010080 (PMC12838048; doi:10.3390/biomedicines14010080)
Supplement: Supplementary file 1 [file biomedicines-14-00080-s001.zip › biomedicines-4004303-supplementary.pdf]

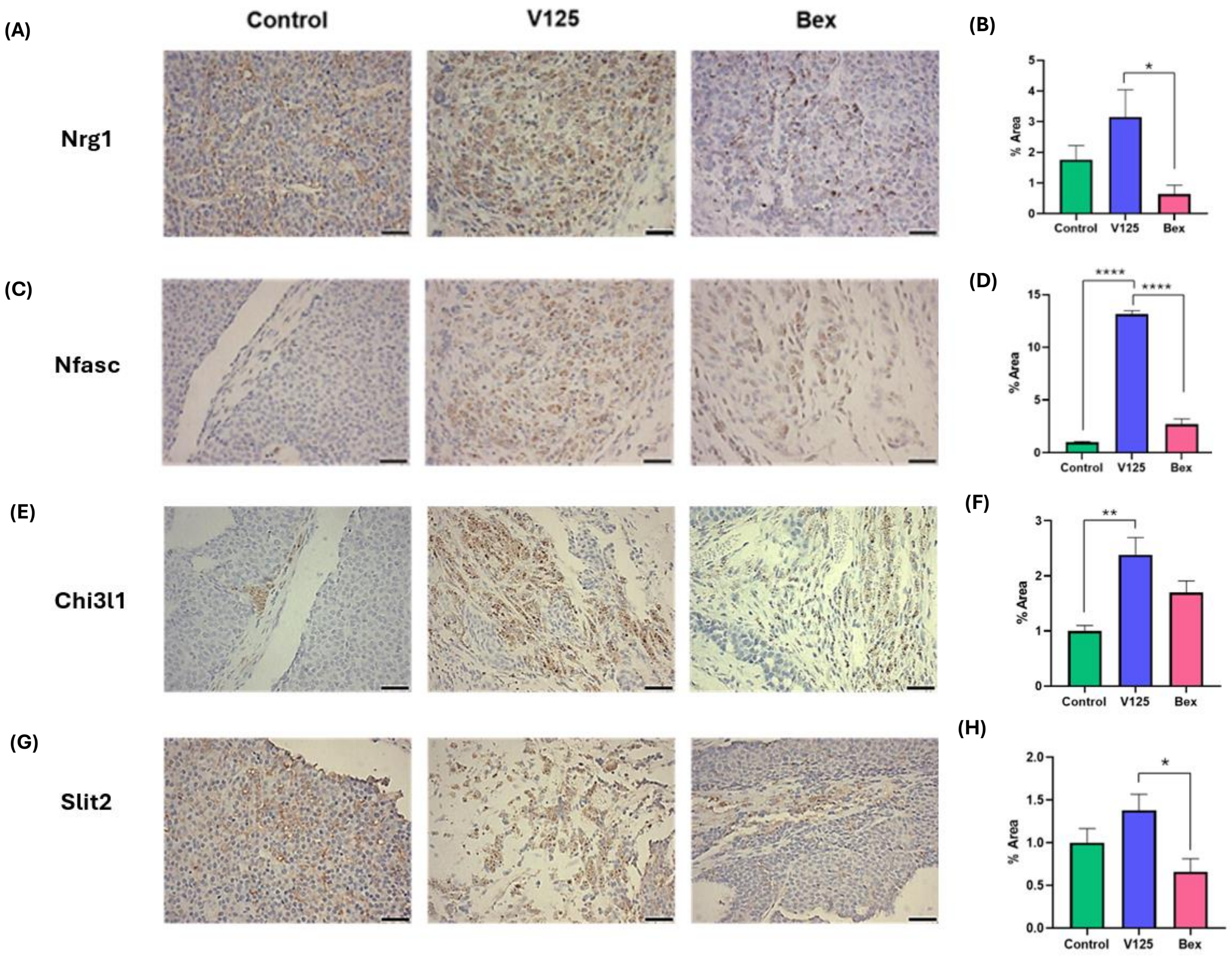

Supplement Figure 1. Differential regulation of cancer and immune response related gene expression by V-125 and bexarotene in MMTV-Neu mammary tumors. (A, C, E, G) Representative IHC images showing the expression of Nrg1, Nfasc, Chi3l1, and Slit2 in tumor tissues from control, V-125-treated, and bexarotene-treated mice. **(B, D, F, H)** Corresponding quantification bar graphs display the percentage of positive staining area (% Area) for each marker across treatment groups. Data are presented as mean ± SEM. **p* < 0.05, ***p* < 0.01, *****p* < 0.0001; n ≥ 3 mice per group. Scale bars = 40 μm. Bex=Bexarotene.

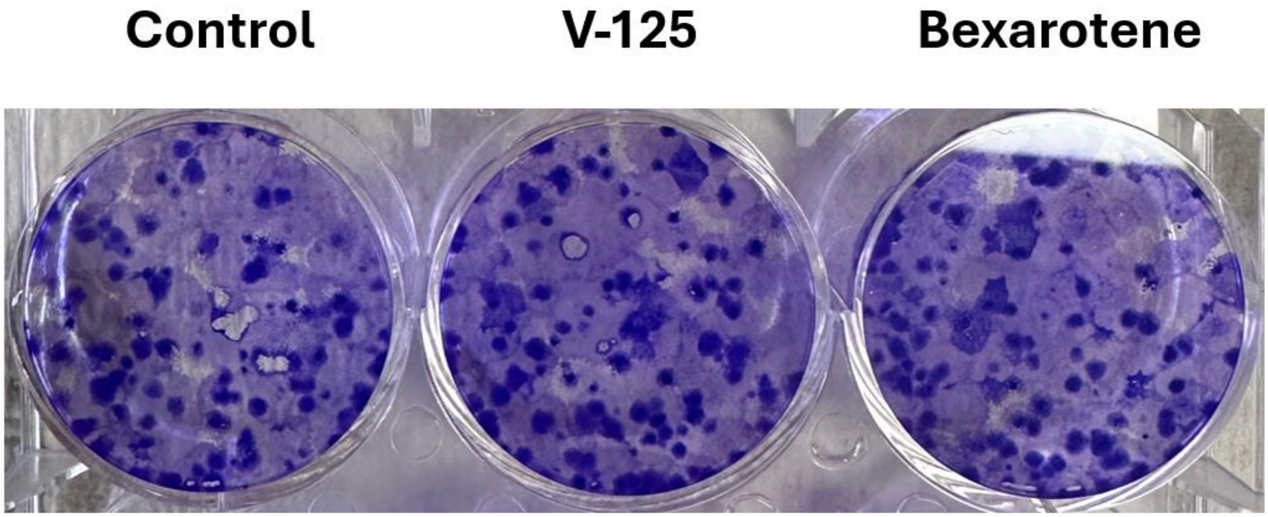

Supplement Figure 2. Effects of V-125 and bexarotene on clonogenic growth in E18-14C-27 $HER2^{+}$ breast cancer cells. Representative images of colony formation in E18-14C-27 cells treated with vehicle control, V-125 (600 nM), or bexarotene (600 nM). Cells were seeded at 500 cells per well in 6-well plates. After 14 days, colonies were fixed and stained with crystal violet to visualize surviving proliferative colonies.

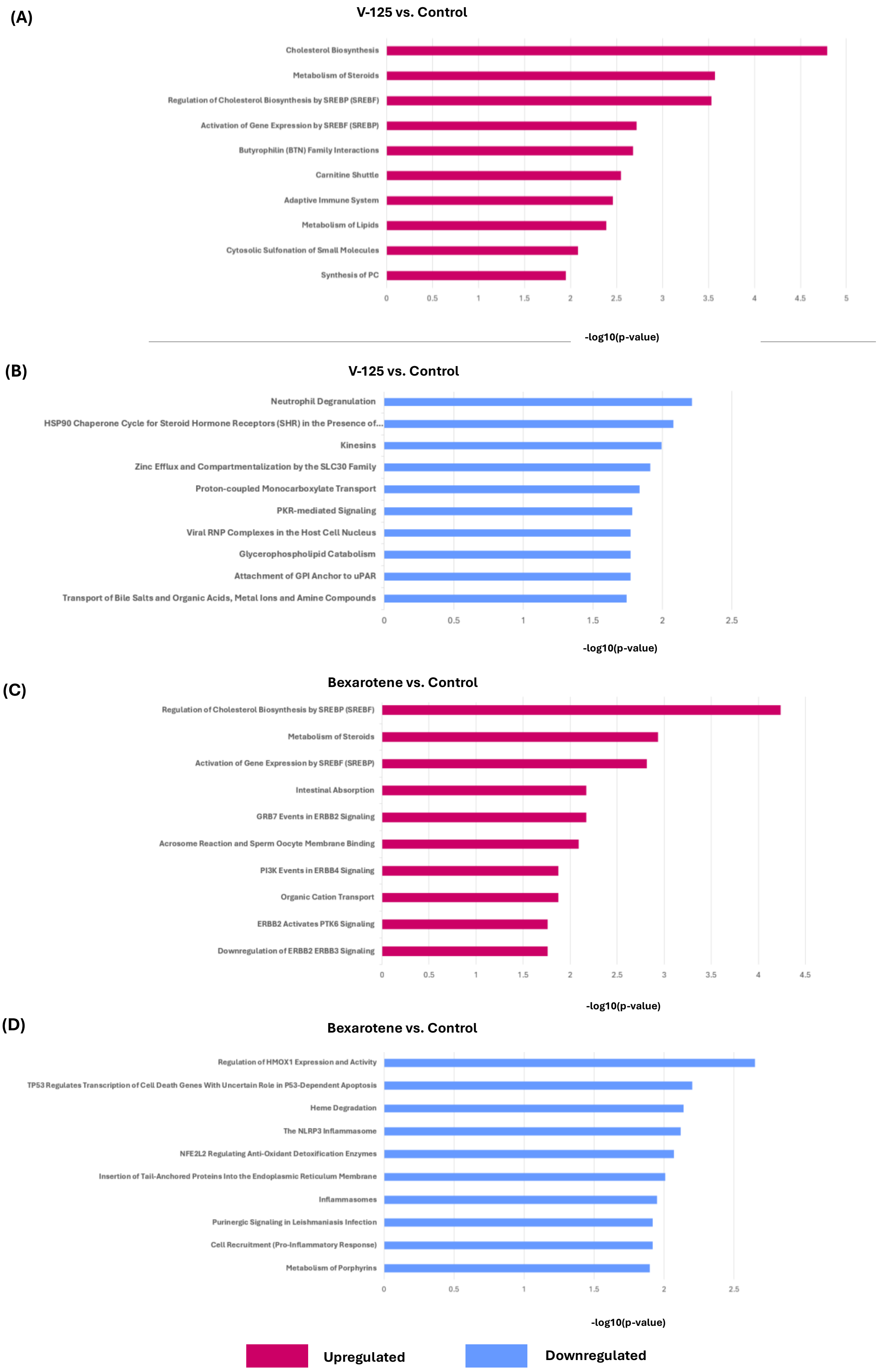

(A)
V-125 vs. Control
Cholesterol Biosynthesis
Metabolism of Steroids
Regulation of Cholesterol Biosynthesis by SREBP (SREBF)
Activation of Gene Expression by SREBF (SREBP)
Butyrophilin (BTN) Family Interactions
Carnitine Shuttle
Adaptive Immune System
Metabolism of Lipids
Cytosolic Sulfonation of Small Molecules
Synthesis of PC
0 0.5 1 1.5 2 2.5 3 3.5 4 4.5 5
-log10(p-value)
(B)
V-125 vs. Control
Neutrophil Degranulation
HSP90 Chaperone Cycle for Steroid Hormone Receptors (SHR) in the Presence of...
Kinesins
Zinc Efflux and Compartmentalization by the SLC30 Family
Proton-coupled Monocarboxylate Transport
PKR-mediated Signaling
Viral RNP Complexes in the Host Cell Nucleus
Glycerophospholipid Catabolism
Attachment of GPI Anchor to uPAR
Transport of Bile Salts and Organic Acids, Metal Ions and Amine Compounds
0 0.5 1 1.5 2 2.5
-log10(p-value)
(C)
Bexarotene vs. Control
Regulation of Cholesterol Biosynthesis by SREBP (SREBF)
Metabolism of Steroids
Activation of Gene Expression by SREBF (SREBP)
Intestinal Absorption
GRB7 Events in ERBB2 Signaling
Acrosome Reaction and Sperm Oocyte Membrane Binding
PI3K Events in ERBB4 Signaling
Organic Cation Transport
ERBB2 Activates PTK6 Signaling
Downregulation of ERBB2 ERBB3 Signaling
0 0.5 1 1.5 2 2.5 3 3.5 4 4.5
-log10(p-value)
(D)
Bexarotene vs. Control
Regulation of HMOX1 Expression and Activity
TP53 Regulates Transcription of Cell Death Genes With Uncertain Role in P53-Dependent Apoptosis
Heme Degradation
The NLRP3 Inflammasome
NFE2L2 Regulating Anti-Oxidant Detoxification Enzymes
Insertion of Tail-Anchored Proteins Into the Endoplasmic Reticulum Membrane
Inflammasomes
Purinergic Signaling in Leishmaniasis Infection
Cell Recruitment (Pro-Inflammatory Response)
Metabolism of Porphyrins
0 0.5 1 1.5 2 2.5
-log10(p-value)
Upregulated
Downregulated

Supplement Figure 3. V-125 and bexarotene enrich distinct pathways in tumors identified by Reactome Pathway 2024 analysis. Bar graphs display the top differentially enriched pathways identified by Reactome Pathway 2024 analysis in Enrichr: **(A)** Upregulated pathways altered in V-125-treated vs. control tumors, **(B)** Downregulated pathways altered in V-125-treated vs. control tumors, **(C)** Upregulated and downregulated pathways altered in bexarotene-treated vs. control tumors and **(D)** Downregulated pathways altered in bexarotene-treated vs. control tumors. Pathway enrichment is plotted as -log10(p-value). Pathways are ranked by significance.

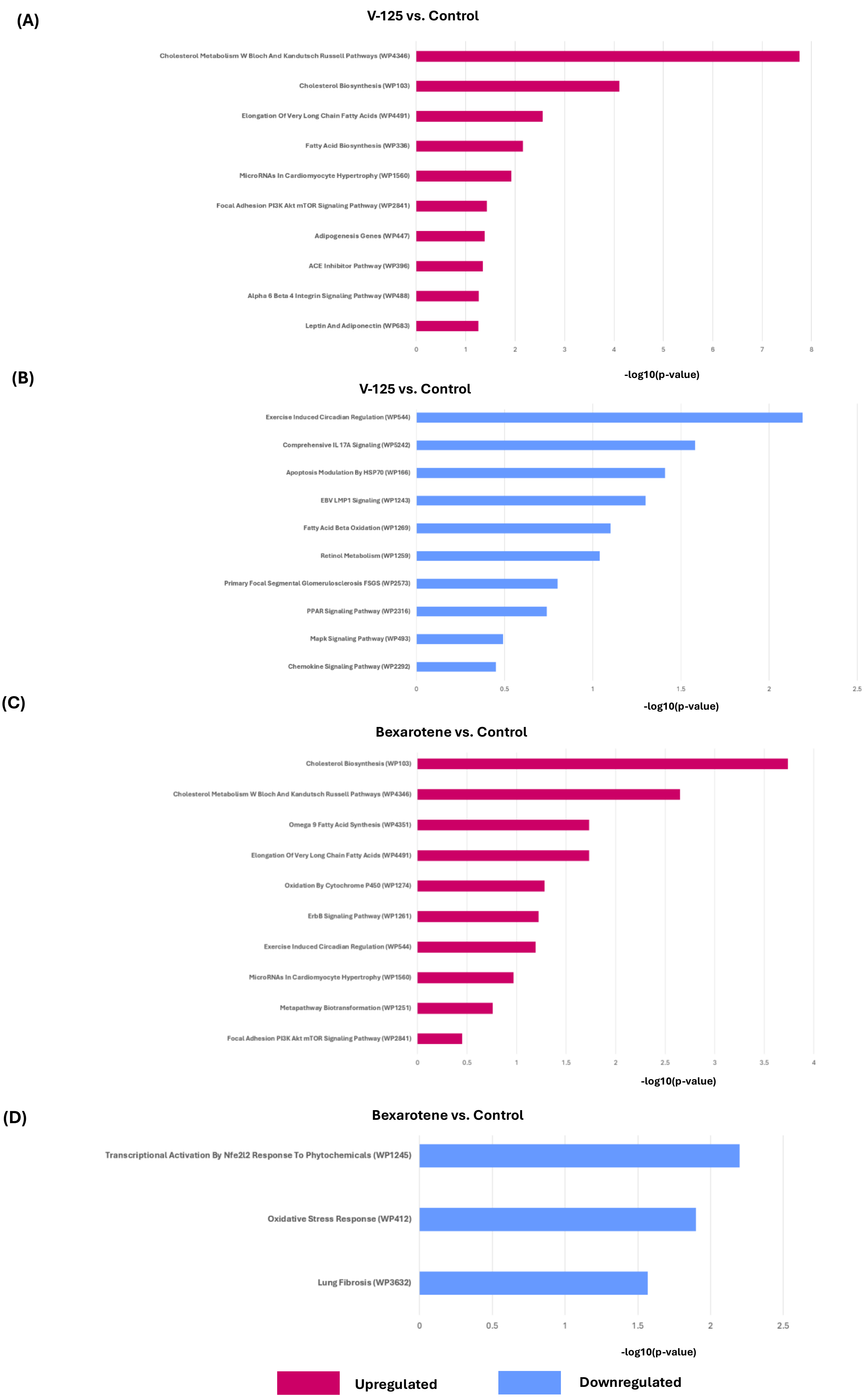

(A)
V-125 vs. Control
Cholesterol Metabolism W Bloch And Kandutsch Russell Pathways (WP4346)
Cholesterol Biosynthesis (WP103)
Elongation Of Very Long Chain Fatty Acids (WP4491)
Fatty Acid Biosynthesis (WP336)
MicroRNAs In Cardiomyocyte Hypertrophy (WP1560)
Focal Adhesion PI3K Akt mTOR Signaling Pathway (WP2841)
Adipogenesis Genes (WP447)
ACE Inhibitor Pathway (WP396)
Alpha 6 Beta 4 Integrin Signaling Pathway (WP488)
Leptin And Adiponectin (WP683)
0
1
2
3
4
5
6
7
8
-log10(p-value)
(B)
V-125 vs. Control
Exercise Induced Circadian Regulation (WP544)
Comprehensive IL 17A Signaling (WP5242)
Apoptosis Modulation By HSP70 (WP166)
EBV LMP1 Signaling (WP1243)
Fatty Acid Beta Oxidation (WP1269)
Retinol Metabolism (WP1259)
Primary Focal Segmental Glomerulosclerosis FSGS (WP2573)
PPAR Signaling Pathway (WP2316)
Mapk Signaling Pathway (WP493)
Chemokine Signaling Pathway (WP2292)
0
0.5
1
1.5
2
2.5
-log10(p-value)
(C)
Bexarotene vs. Control
Cholesterol Biosynthesis (WP103)
Cholesterol Metabolism W Bloch And Kandutsch Russell Pathways (WP4346)
Omega 9 Fatty Acid Synthesis (WP4351)
Elongation Of Very Long Chain Fatty Acids (WP4491)
Oxidation By Cytochrome P450 (WP1274)
ErbB Signaling Pathway (WP1261)
Exercise Induced Circadian Regulation (WP544)
MicroRNAs In Cardiomyocyte Hypertrophy (WP1560)
Metapathway Biotransformation (WP1251)
Focal Adhesion PI3K Akt mTOR Signaling Pathway (WP2841)
0
0.5
1
1.5
2
2.5
3
3.5
4
-log10(p-value)
(D)
Bexarotene vs. Control
Transcriptional Activation By Nfe2l2 Response To Phytochemicals (WP1245)
Oxidative Stress Response (WP412)
Lung Fibrosis (WP3632)
0
0.5
1
1.5
2
2.5
-log10(p-value)
Upregulated
Downregulated

Supplement Figure 4. V-125 and bexarotene enrich distinct pathways in tumors identified by WikiPathways 2024 Mouse analysis. Bar graphs display the top differentially enriched pathways identified by WikiPathways 2024 Mouse analysis in Enrichr: **(A)** Upregulated pathways altered in V-125-treated vs. control tumors, **(B)** Downregulated pathways altered in V-125-treated vs. control tumors, **(C)** Upregulated and downregulated pathways altered in bexarotene-treated vs. control tumors and **(D)** Downregulated pathways altered in bexarotene-treated vs. control tumors. Pathway enrichment is plotted as -log10(p-value). Pathways are ranked by significance.
